# Composition-tunable transition metal dichalcogenide nanosheets *via* a scalable, solution-processable method†

**DOI:** 10.1039/d3nh00477e

**Published:** 2024-01-30

**Authors:** Rebekah A. Wells, Nicolas J. Diercks, Victor Boureau, Zhenyu Wang, Yanfei Zhao, Simon Nussbaum, Marc Esteve, Marina Caretti, Hannah Johnson, Andras Kis, Kevin Sivula

**Affiliations:** a Laboratory for Molecular Engineering of Optoelectronic Nanomaterials, Institute of Chemical Sciences and Engineering, École Polytechnique Fédérale de Lausanne (EPFL) CH-1015 Lausanne Switzerland Rebekah.Wells@epfl.ch Kevin.Sivula@epfl.ch; b Interdisciplinary Center for Electron Microscopy (CIME), École Polytechnique Fédérale de Lausanne (EPFL) CH-1015 Lausanne Switzerland; c Laboratory of Nanoscale Electronics and Structures, Institute of Materials Science and Engineering, École Polytechnique Fédérale de Lausanne (EPFL) CH-1015 Lausanne Switzerland; d Advanced Materials Research, Toyota Motor Europe B-1930 Zaventem Belgium

## Abstract

The alloying of two-dimensional (2D) transition metal dichalcogenides (TMDs) is an established route to produce robust semiconductors with continuously tunable optoelectronic properties. However, typically reported methods for fabricating alloyed 2D TMD nanosheets are not suitable for the inexpensive, scalable production of large-area (m^2^) devices. Herein we describe a general method to afford large quantities of compositionally-tunable 2D TMD nanosheets using commercially available powders and liquid-phase exfoliation. Beginning with Mo_(1−*x*)_W_*x*_S_2_ nanosheets, we demonstrate tunable optoelectronic properties as a function of composition. We extend this method to produce Mo_0.5_W_0.5_Se_2_ MoSSe, WSSe, and quaternary Mo_0.5_W_0.5_SSe nanosheets. High-resolution scanning transmission electron microscopy (STEM) imaging confirms the atomic arrangement of the nanosheets, while an array of spectroscopic techniques is used to characterize the chemical and optoelectronic properties. This transversal method represents an important step towards upscaling tailored TMD nanosheets with a broad range of tunable optoelectronic properties for large-area devices.

## Abstract 2

Alloying is a powerful tool for fine tuning the optoelectronic properties of 2D TMDs. We present a general, solution-processable route for the scalable production of large quantities of alloyed 2D TMD nanosheets from commercially available powders.

## Graphical abstract

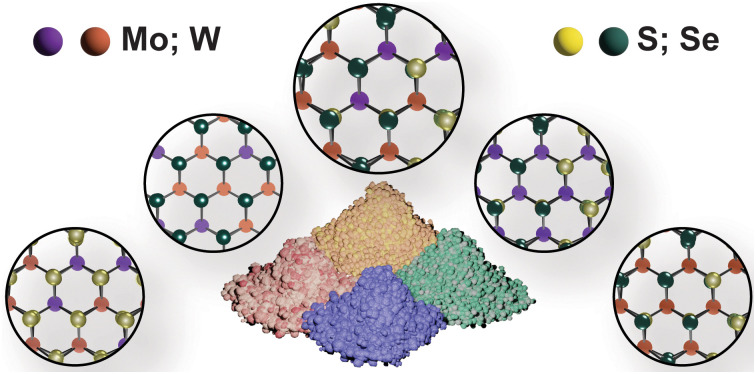

**New concepts**

Alloyed two-dimensional (2D) transition metal dichalcogenides (TMDs) are an important emerging class of optoelectronic materials. In particular, the ability to precisely tune their composition and therefore optoelectronic properties is highly desirable. While previous works have demonstrated this ability using bottom-up approaches such as chemical vapor deposition/transport (CVD/CVT) or molecular beam epitaxy (MBE), these methods are not suitable for inexpensive, large scale production of nanomaterials. To fill this gap, we introduce a top-down, solution processable production method capable of enabling compositional control over 2D TMD alloys while also being amenable to large scale production. As a result, this facilitates large area (m^2^) film fabrication on a variety of rigid and flexible substrates and opens new avenues for other applications such as additive manufacturing, composite materials, or photocatalytic particle systems. Furthermore, our technique is readily adapted to produce a variety of ternary and quaternary alloys based on Mo, W, S, and Se. In summary, this work establishes a new concept for the sustainable production of alloyed 2D TMDs for optoelectronic applications.

Layered van der Waals materials such as transition metal dichalcogenides (TMDs) are promising candidates for electronic and optoelectronic applications thanks to their robust nature and versatility. With nearly 40 different MX_2_ (M = metal, X = chalcogen) combinations, TMDs span the full spectrum of electronic materials from semimetal to semiconductor and superconductor.^1^ Moreover, given the similar crystal structures of the MX_2_ TMDs, substituting different metals or chalcogens into a pure material by doping or alloying can further expand the tunability of this class of materials.^2^ Indeed, early research efforts demonstrated a number of possible combinations through the growth of single crystal and polycrystalline ternary alloys such as MoWSe_2_ and MoWTe_2_.^3,4^ These seminal studies showed that, for the same annealing temperature, some compounds were completely miscible (*i.e.*, MoSe_2_, WSe_2_), while others (MoTe_2_, WTe_2_) possessed solubility limits.^3^ The more recent development of two-dimensional (2D) TMD materials, which provides new levels of property control based on the number of atomic layers and their heterojunctions, has led to a resurgence in reports of TMD alloys with heightened interest for applications in electronics, catalysis, sensing, and optoelectronics.^5–12^

Contemporary studies have demonstrated various alloyed 2D TMDs by leveraging bottom-up techniques such as chemical vapor deposition (CVD),^13,14^ chemical vapor transport (CVT),^12^ atomic layer deposition (ALD),^15^ molecular beam epitaxy (MBE),^16^ or else CVD in combination with plasma or laser modification.^10,17–19^ These methods have been instrumental for fundamental studies of these materials as well as demonstrating alloying as a viable avenue for fine tuning the optoelectronic properties *via* bandgap engineering. However, they generally use energy intensive and multistep growth cycles, require specific growth substrates, depend on custom-made apparatuses, and involve subsequent material transfer methods for device fabrication, rendering these methods generally unscalable beyond the centimeter length-scale. Furthermore, these methods are not amenable to applications which would require large volumes of nanosheet dispersions (*e.g.* for composite materials, additive manufacturing, photocatalytic particle systems, *etc.*).^20,21^ Given the demonstrated promise of alloyed 2D TMD nanomaterials, it is necessary to develop methods to produce large quantities of materials that are amenable to a variety of processing techniques and which can be used for large-area (m^2^–km^2^) optoelectronic devices (*e.g.*, solar energy conversion).^20–23^

The liquid-phase exfoliation (LPE) of bulk alloyed material into alloyed nanoflakes, represents a promising route to overcome the challenge of large-scale production. While using ultrasonication^6,11^ as an exfoliation technique has been demonstrated, this method produces nanoflakes with high defect concentration and low aspect ratios, which is unsuitable for high-performance optoelectronic applications.^11,24,25^ Thus, there remains a critical need to develop techniques that can both produce large quantities of nanomaterial while maintaining the ability to control the composition of the nanosheets.

Herein we present a solution-processable route for producing nanosheets of 2D TMD alloys. Our method uses inexpensive, commercially available starting materials and leverages an electrochemical pellet intercalation (ECPI) technique which has been shown to produce pure-phase 2D TMD nanosheets with low defect concentrations compared to traditional LPE approaches (*e.g.*, ultrasonication).^24^ We show that this method can be used to make a range of TMD alloys (M = Mo, W; X = S, Se), including ternary and quaternary alloys, with precise control over the composition. Importantly similar material properties are observed in our solution-processed nanosheets as in those made *via* CVD, ALD, or MBE, suggesting alloyed TMD nanosheets can become an economically viable component for incorporation into next-generation, large-area (m^2^–km^2^) optoelectronic and electronic devices.

Fig. 1 schematically shows our route to prepare alloyed 2D TMD nanosheets. Briefly, two pure TMD bulk powders are mechanically mixed in the desired ratio, pressed, and annealed in a sealed quartz tube to form a conductive polycrystalline pellet. Note that all starting materials and ratios are conserved. Annealing conditions are chosen based on the chalcogen species (higher temperatures are required for S-based TMDs compared to Se^26^). For example, S-based compounds are heated to 1100 °C and held for 24 hours *versus* 1000 °C for 12 hours for Se-based materials. Electrochemical intercalation of tetraheptylammonium (THA^+^), gentle exfoliation *via* bath sonication, and low-speed centrifugation (120 rcf) are performed to isolate a dispersion of alloyed 2D TMD nanosheets. Note that this dispersion contains a range of flake thicknesses from mono- to few-layer and different flake sizes can be selected according to the needs of the intended application.^23,27,28^ For complete details on the annealing, electrochemical intercalation, exfoliation, and size-selection conditions, please see the Experimental methods section in the ESI.†

**Fig. 1:**
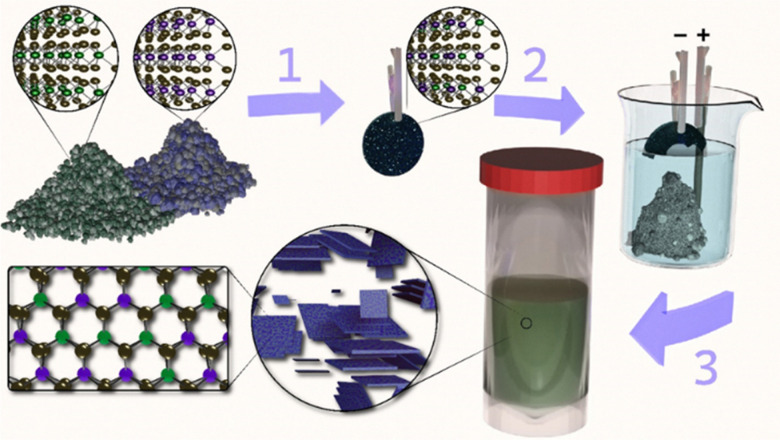
Scheme to form 2D TMD alloy nanosheets. (1) As-received TMD powders are mechanically mixed, pressed into a pellet, and annealed (*e.g.* at 1100 °C for 48 h for MoS_2_ and WS_2_). (2) The annealed pellet acts as the cathode in tetraheptylammonium bromide (THA^+^ Br^−^) electrolyte with a glassy carbon anode. THA^+^ intercalation (driven by an applied bias) leads to the eventual detachment of intercalated material. (3) The intercalated powder is collected, rinsed, gently agitated, and centrifuged at low speed to remove remaining bulk material. The result is a concentrated solution of exfoliated, alloyed 2D TMD nanosheets.

As a first demonstration, the Mo_(1−*x*)_W_*x*_S_2_ system was chosen as it is one of the most well-studied ternary alloys to date.^6,10–13,15,19,29–33^ For *x* = 0.5, after confirming the formation of the Mo_0.5_W_0.5_S_2_ alloy in the annealed pellet *via* X-ray diffraction (XRD) (see Fig. S1, ESI†), and performing the exfoliation, the resulting dispersion was characterized in dispersion or as nanosheet thin films made by a previously-described liquid–liquid interfacial self-assembly (LLISA) method,^34^ which has also been demonstrated as a reliable route for roll-to-roll large-area (m^2^) film fabrication.^35^ Furthermore, this film formation technique was used to deposit the alloyed nanosheets on a variety of rigid and flexible substrates (glass, Au-patterned Si/SiO_2_, glass beads, PET, Fig. S2, ESI†).

At the nanometer scale, X-ray photoelectron spectroscopy (XPS) confirmed the presence of all of the atomic species in thin films of nanosheets (Fig. S3, ESI†), and elemental mapping of individual nanosheets by scanning transmission electron microscopy (STEM) energy dispersive X-ray (EDX) spectroscopy demonstrated the homogeneous distribution of metal atoms over a *ca.* 500 nm nanosheet as shown in Fig. 2a. This suggests the successful formation of the 2D alloy at the nanometer scale. We note that thorough mechanical mixing before pellet formation is required (See Experimental methods section in the ESI†); in contrast to Fig. 2a, pure binary phase nanosheets (*i.e.*, MoS_2_ and WS_2_) could be observed in poorly mixed exfoliated samples (See Fig. S4, ESI†).

**Fig. 2:**
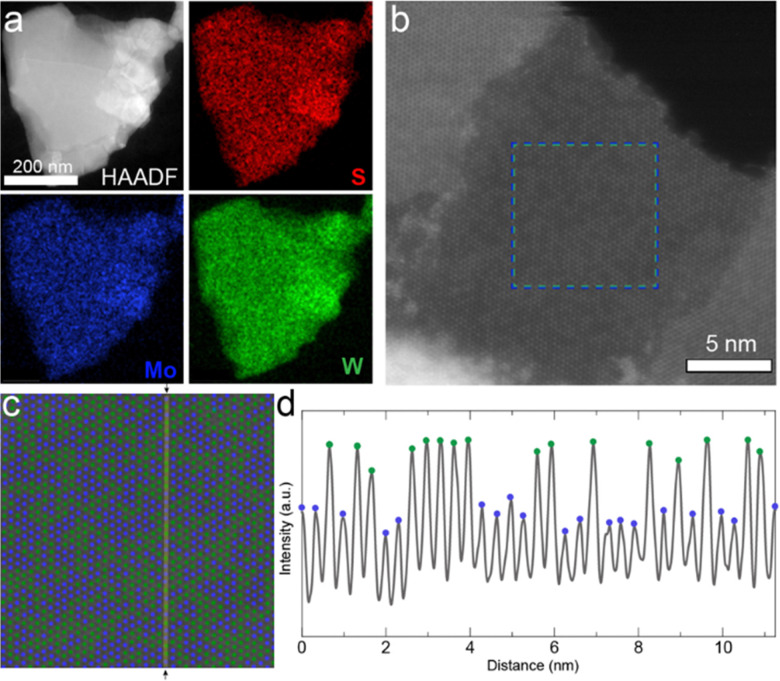
STEM analysis of Mo_0.5_W_0.5_S_2_ nanosheets. (a) STEM HAADF imaging and EDX elemental maps of a typical nanosheet show presence of S (top right), Mo (bottom left), and W (bottom right) distributed across the flake. (b) Atomic-resolution STEM HAADF image of monolayer region of a Mo_0.5_W_0.5_S_2_ nanosheet. Brighter atom contrasts correspond to W; darker atom contrasts to Mo; S is not visible. (c) Cropped and enlarged region highlighted in (b), where W atoms were colored in green and Mo in blue. (d) Line profile of the HAADF intensity as indicated in (c), along the *a*-axis [110] of the crystal. The maxima correspond to metal atoms and minima to vacuum in-between the atoms.

To confirm the sub-nanometer distribution of the Mo and W atoms in the well-mixed sample, high-resolution STEM high-angle annular dark-field (HAADF) was performed on a monolayer region of a Mo_0.5_W_0.5_S_2_ nanosheet as shown in Fig. 2b. HAADF imaging can be used to identify atoms of different atomic number (*Z*), such as Mo and W, as the intensity of the signal scales according to ∼*Z*^2^.^14^ W is the heaviest atom and thus interacts most strongly with the electron beam, appearing brighter compared to Mo (Fig. 2c and d). However, it is not possible to precisely observe the S atoms with this technique as its *Z* is too small relative to the metal atoms. For better visualization, the Mo and W atoms are depicted in blue and green, respectively, in Fig. 2c. An intensity profile of the HAADF image along the *a*-axis [110] is plotted in Fig. 2d. The maxima associated to W and Mo atom positions can be easily distinguished with the higher maxima belonging to W atoms and the lower maxima to Mo atoms. The fluctuation in the minima, between the atoms, is explained by the different chemical environments in the vicinity of the probe position in the image due to the spatial resolution of the STEM measurement. Generally speaking, the minimum shows a higher signal when surrounded by W atoms. For comparison, additional high-resolution STEM HAADF images of alloyed nanosheets can be found in Fig. S5 (ESI†).

Notably, the distribution of the metal atoms appears disordered. While preliminary computational works had suggested that the most energetically favorable atomic configurations are those in which Mo–S–W bonds are maximized giving an ordered W/Mo/W pattern,^29^ experimental works exclusively show a disordered phase, or a clustering of atoms (*i.e.* two or more adjacent same-element atoms).^10,12,14,31^ Indeed, Tan and coworkers re-examined this phenomenon and found that the disordered phase becomes increasingly favorable with increasing temperature.^36^ Therefore, given the annealing conditions used in this work (1100 °C), our observation of a disordered phase is consistent with both recent computational and experimental work.

The high-resolution STEM HAADF images also allows a precise calculation of the W : Mo atomic ratio of the monolayer region and comparison to the intended 1 : 1 atomic ratio of the pellet. In the exfoliated nanosheet shown in Fig. 2c, 710 W atoms and 642 Mo atoms were identified over the 1352 atoms displayed, yielding a W : Mo ratio of 1.11 : 1 (53 at% W, based on metal). Additional nanosheets from the same batch were examined, giving W ranging from 46 to 54 at%. The accordance between the atomic feed ratio and the composition of the resulting TMD nanosheets suggests that compositional control can be exerted over the alloyed nanosheets with this alloying/exfoliation process.

To confirm that precise control over nanosheet atomic composition is accessible with our method, alloyed 2D nanosheets with a range of metal feed ratio were prepared and their properties examined as a function of composition. Fig. 3a shows the Raman spectra for LLISA deposited 2D nanosheet films of MoS_2_, Mo_0.6_W_0.4_S_2_, Mo_0.5_W_0.5_S_2_, Mo_0.4_W_0.6_S_2_, and WS_2_. Pure MoS_2_ displays the expected peaks at 385 cm^−1^ (E^1^_2g_) and 410 (A_1g_) consistent with few-layer MoS_2_.^15^ With increasing W content, the A_1g_ mode shifts to higher wavenumbers, eventually reaching 422 cm^−1^ for pure, few-layer WS_2_, but generally retains its shape and character, consistent with computational predictions and previous experimental results.^15,37,38^ In contrast the E^1^_2g_ peak from pure MoS_2_ shifts to lower wavenumbers and splits into two separate peaks in the alloyed nanosheets, corresponding to S–W–S and S–Mo–S in-plane vibrational modes.^37^ For pure WS_2_, one peak is once again observed for E^1^_2g_ at 350 cm^−1^, in line with previous studies.^15,37,38^ These trends are consistent with theoretical predictions and those observed in Mo_(1−*x*)_W_*x*_S_2_ nanosheets prepared by CVD or ALD,^10,15,39,40^ thereby confirming compositional control in the nanosheet thin films made using the method presented here.

**Fig. 3:**
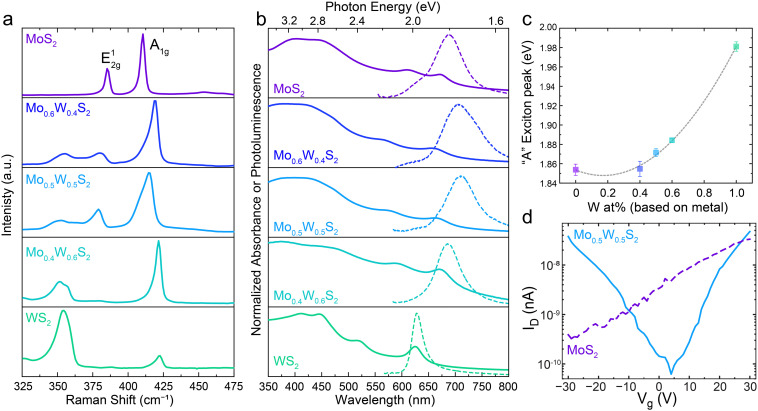
Investigation of optoelectronic properties of Mo_(1−*x*)_W_*x*_S_2_ nanosheets for *x* = 0 (purple, top), *x* = 0.4 (blue), *x* = 0.5 (light blue), *x* = 0.6 (teal), and *x* = 1 (green, bottom). (a) Raman spectroscopy of a LLISA deposited thin film of 2D Mo_(1−*x*)_W_*x*_S_2_. (b) Normalized UV-Vis absorbance spectra (solid lines) for Mo_(1−*x*)_W_*x*_S_2_ nanosheet dispersions and normalized PL (broken lines) for thin films. (c) Extracted energies of the A excitonic peaks from the UV-Vis spectra (solid lines in panel b) plotted as a function of W atomic content, *x*. The black solid curve is the quadratic fit for the extracted data points and describes the bowing effect observed. (d) Transfer curves for FETs made up of pure MoS_2_ (purple, broken line) and alloyed Mo_0.5_W_0.5_S_2_ (blue, solid line) for a source-drain voltage of 30 V.

Next, the optoelectronic properties were evaluated as a function of composition. Fig. 3b shows the normalized ultraviolet visible (UV-Vis) absorbance spectra for the Mo_(1−*x*)_W_*x*_S_2_ dispersions (solid lines) and the photoluminescence (PL) of the films (broken lines) studied in Fig. 3b. Once again, the pure materials display the expected excitonic peaks at 671 nm (A) and 609 nm (B) for MoS_2_ (top) and 625 nm (A) for WS_2_ (bottom).^41^ Importantly, distinct excitonic peaks are present in the alloyed nanosheet dispersions, as opposed to a mixture of MoS_2_ and WS_2_ signals. To better visualize their relationship, the “A” exciton peak energies from each UV-vis spectrum in Fig. 3b have been extracted and plotted in Fig. 3c, allowing for visualization of the trend in the optical bandgap as a function of alloy composition. Fitting these points yields a parabolic relationship, demonstrating a well-studied phenomenon known as “band bowing” wherein the bandgap energy varies parabolically as a function of alloy composition.^12,14,15,29,30,39,40,42^ Indeed, a bowing parameter of 0.19 ± 0.01 is calculated, which is in excellent agreement with previous studies based on CVD and ALD-made monolayers.^10,12,15^

In accordance with the absorbance spectra, the bowing trend can be seen in the PL spectra of the thin films (Fig. 3b, broken lines). It should be noted that the UV-Vis absorbance spectra give an overview of all the nanosheets present in solution (ensemble measurement) while the PL focuses on a small region of a single film. Notably, PL can be recorded for each alloy mixture, though intensity, Stokes shift, and peak sharpness can vary as a result of the flake morphology in the region of interest as previously observed.^24^ Indeed, additional tuning is possible by controlling layer number, which in our method can be tuned during the centrifugation step, presenting an advantage over CVD-based preparation methods when multi-layered flakes are desired.^15,43^ Together these results indicate that both absorbance and emission can be fine-tuned using our powder alloying method.

To observe differences in trends in the electronic behavior, bottom gate field-effect transistors (FETs) were made using pure MoS_2_ or alloyed Mo_0.5_W_0.5_S_2_ nanosheets as the active semiconducting material. Representative transfer curves can be seen in Fig. 3d and the related output curves can be found in Fig. S6 (ESI†). We note that this method relies on nanosheets bridging the channel gap in such a way that a charge percolation path is created, and that this path is not always the most direct, leading to high contact resistances and making it difficult to reliably extract the charge mobilities for quantitative comparison.

For the MoS_2_ devices (purple, broken line), only the typical^24,43–45^ n-type behavior is observed, while the Mo_0.5_W_0.5_S_2_ devices (light blue, solid line) display ambipolar behavior. Previous theoretical work suggests that this could be the result of a decrease in the effective mass of holes, allowing for improved hole mobility compared to either pure material.^33^ Furthermore, the use of high work function metal contacts (gold) readily accommodates injection of both electrons and holes, allowing for the observation of ambipolar behavior, which might not have been possible with other lower work function metals.^33^ Though not considered in this work, impurities or defects could also affect the conduction polarity and are of particular interest for future works.

The ambipolar behavior displayed by the alloyed Mo_0.5_W_0.5_S_2_ nanosheets qualitatively confirms unique electronic properties compared to the pure MoS_2_ nanosheets. This suggests that electronic behavior, in addition to optical and optoelectronic, can be modulated using this powder-based scalable method.

Given the success of producing compositionally controlled Mo_(1−*x*)_W_*x*_S_2_ nanosheets using the ECPI method, we next confirmed the adaptability of the method by preparing all of the remaining possible ternary alloy combinations using Mo, W, S, and Se: Mo_0.5_W_0.5_Se_2_, MoSSe, and WSSe. XPS spectra of thin films of the alloyed nanosheets confirms the presence of the atomic species according to the alloy composition (Fig. S3, ESI†) while Raman spectra (Fig. S7, ESI†) confirm alloy formation with the appearance of unique vibration modes consistent with monolayer CVD-grown demonstrations.^7,8,46–48^ Unique optoelectronic properties for each ternary alloy were recorded *via* UV-Vis and PL as shown in Fig. S7 (ESI†). Importantly this confirms the possibility to tune both the metal (Mo_(1−*x*)_W_*x*_S_2_, Mo_(1−*x*)_W_*x*_Se_2_) and chalcogen (MoS_(1−*y*)_Se_*y*_, WS_(1−*y*)_Se_*y*_) atom concentration using this one general method.

Based on the results above, quaternary alloyed Mo_(1−*x*)_W_*x*_S_(2−*y*)_Se_*y*_ nanosheets can be accessed either by mixing all four binary TMD powders (MoS_2_, WSe_2_, WS_2_, MoSe_2_), or simply mixing MoS_2_ with WSe_2_ in equimolar amounts to obtain Mo_0.5_W_0.5_SSe nanosheets. Successful alloying of a Mo_0.5_W_0.5_SSe pellet was confirmed *via* XRD (Fig. S8, ESI†) followed by ECPI assisted exfoliation to produce the quaternary alloyed nanosheets. XPS of nanosheet films was used to confirm the presence of all four elements (Fig. S9, ESI†) while elemental distribution and atomic configuration was investigated using STEM. Fig. 4a shows STEM EDX elemental maps of a Mo_0.5_W_0.5_SSe nanosheet suggesting the presence of all four elements with homogenous distribution at the nanometer scale. High-resolution STEM was used to probe distribution at the atomic scale. HAADF (Fig. 4b) images show a contrast for the heavier metal atoms while STEM integrated differential phase contrast (iDPC) imaging also allows for the visualization of the lighter chalcogen atoms (Fig. 4c). Note that iDPC images show a ∼linear intensity scaling with *Z*, as opposed to *Z*^2^ for HAADF, making this latter visualization possible.^49,50^

**Fig. 4:**
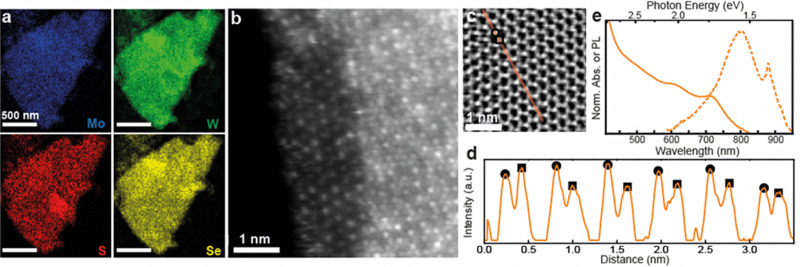
Analysis of ternary 2D nanosheets. (a) STEM EDX elemental maps of a typical nanosheet shows presence of Mo (top left), W (top right), S (bottom left), and Se (bottom right) distributed across the flake. (b) Atomic-resolution STEM HAADF image of the edge of a multilayer Mo_0.5_W_0.5_SSe nanosheet. The left area is monolayer and shows W atoms (brighter) and Mo atoms (dimmer). The right area is trilayer. (c) Atomic-resolution STEM iDPC image of a trilayer flake where both metal and chalcogen atoms are visible. (d) Line profile of the iDPC intensity, along the *m*-axis [100] of the crystal, for line highlighted in (c), where the higher, on average, intensity maxima (circles) are assigned to the M–X_2_–M columns and the lower, on average, intensity maxima (squares) are assigned to the X_2_–M–X_2_ columns. (e) Normalized UV-Vis absorbance spectra (solid lines) for Mo_0.5_W_0.5_SSe nanosheet dispersions and normalized PL (broken lines) for a thin film.

The HAADF image in Fig. 4b shows the edge of a quaternary alloyed nanosheet, including a small monolayer region (left) and a trilayer region (right). In the monolayer region, the atomic distribution of Mo (dimmer contrast) and W (brighter contrast) atoms is visible and a clear atomic mixing is observed. In the trilayer area, the contrast at each atom column location is influenced by the layering of different combinations of atoms in either a M–X_2_–M or X_2_–M–X_2_ configuration, with metal atoms making the largest contribution according to the ∼*Z*^2^ atom contrast. Despite the complexity, the different column contrasts suggest different atomic combinations and also supports atomic mixing of the metal atoms in this region of the specimen. Thus, the atomic scale distribution of the two metal species is in good agreement with STEM EDX measurements. Additionally, the quaternary alloy displays the same atomic clustering (adjacent same-element atoms) recorded in the Mo_0.5_W_0.5_S_2_ specimens. The result is consistent with CVD-grown quaternary alloyed monolayers.^14^

To observe the lighter chalcogen atoms, an iDPC image of a trilayer region of a Mo_0.5_W_0.5_SSe nanosheet is shown in Fig. 4c. The hexagonal atomic lattice of the nanosheet is observed along the *c*-axis, consistent with the expected 2H polymorph.^1^ A line profile of the iDPC intensity along the *m*-axis [100] is shown in Fig. 4d, for the orange line highlighted in Fig. 4c. From this profile a periodic pattern is observed with two types of maxima corresponding to heavier atom columns (circles) and lighter atom columns (squares). Heavier atom columns are composed of two M-sites and one X_2_-site (M–X_2_–M), while lighter atom columns are composed of one M-site and two X_2_-sites (X_2_–M–X_2_), where all atomic species significantly contribute according to the ∼*Z* atom contrast. In each layer, metal M-sites (Mo or W) have two possible atomic configurations, and chalcogen X_2_-sites (S or Se) have 5 possible atomic combinations (*i.e.*, S–S, S–Se, Se–Se, S-vacancy, Se-vacancy). While the number of possible atomic combinations renders exact identification of the atomic stacking at each site difficult, both the circle- and square-type intensity maxima vary along the line selection, indicating the presence of different combinations of chemical species at each atom column location. As a rough estimation, an average atomic ratio of 52 : 48 for W : Mo and of 43 : 57 for Se : S was measured using the STEM-EDX maps in Fig. 4a. The atomic resolution STEM images support the STEM EDX observations and confirm the formation of the Mo_0.5_W_0.5_SSe alloy.

Alloy formation on a large scale is additionally supported by Raman spectroscopy of a thin film of Mo_0.5_W_0.5_SSe nanosheets (Fig. S9, ESI†). Indeed, the Raman spectrum is complex as is expected for a material with many vibrational modes including: Mo–S–W, W–S, Mo–S, Mo–W–Se, W–S–Se, Mo–S–Se.^14^ Importantly this spectrum is distinct from either pure material as well as from all of the ternary alloys previously discussed (see Fig. S7 for comparison, ESI†).

Finally, the optoelectronic properties are probed *via* UV-Vis of nanosheet dispersions and PL of a thin film (Fig. 4e). The UV-Vis (solid lines) is unique with respect to either starting powder material (MoS_2_ and WSe_2_, see Fig. 3 for comparison) as well as all of the previous ternary alloy blends (see Fig. S7 for comparison, ESI†), while maintaining clear excitonic peaks around 608 nm and 711 nm. A PL signal can be observed around 1.5 eV and confirms the quality of the quaternary alloyed nanosheets. This demonstration paves the way for additional degrees of tunability by allowing for simultaneous adjustment of the transition metal and chalcogen atoms within a single, solution-processed nanosheet.

## Conclusions

In summary, we have presented a simple, solution-processable route for the scalable production of large quantities of 2D TMD alloyed nanosheets. Using commercially available powders and ECPI-assisted exfoliation, we prepared and characterized five different alloy combinations, including four ternary and one quaternary combination. We obtained concentrated dispersions that were transformed into nanosheets films on the cm^2^ scale, using film formation techniques that have been previously demonstrated to be amenable to m^2^ scale and beyond,^35^ and analyzed their composition and optoelectronic properties. Conveniently this method is suitable for metal (Mo_(1−*x*)_W_*x*_X_2_) and chalcogen (MoS_(2−*y*)_Se_*y*_) ternary alloyed nanosheets or both (Mo_(1−*x*)_W_*x*_S_(2−*y*)_Se_*y*_) to give quaternary alloyed nanosheets. Furthermore, we demonstrated that our method can be used to tune the ensemble optic, electronic, and optoelectronic properties as a function of alloy composition. Notably the materials properties observed are consistent with those reported for CVD, MBE, and ALD-made monolayers, thereby representing a suitable alternative that facilitates production of large-quantities of nanomaterial. By using scalable and easily adaptable methods, this work constitutes an important step forward in making large-area devices based on alloyed TMD nanosheets for a diverse range of electronic and optoelectronic applications.

## Author contributions

R.A. Wells: conceptualization, formal analysis, investigation, methodology, validation, visualization, writing – original draft, review & editing. N.J. Dierks: investigation, validation, writing – review & editing. V. Boureau: investigation, formal analysis, validation, writing – review & editing. Z. Wang: investigation, validation, writing – review and editing. Yanfei Zhao: investigation. S. Nussbaum: investigation, validation. M. Esteve: investigation. M. Caretti: investigation, writing – review & editing. H. Johnson: writing – review & editing. A. Kis: supervision, validation, resources. K. Sivula: conceptualization, visualization, resources, supervision, writing – review & editing. The manuscript was written through contributions of all authors. All authors have given approval to the final version of the manuscript.

## Conflicts of interest

The authors declare no competing financial interest.

## Supplementary Material

NH-009-D3NH00477E-s001
