# Supplementary material for: Composition-tunable transition metal dichalcogenide nanosheets via a scalable, solution-processable method
Source: Nanoscale Horiz. 2024 Jan 30;9(4):620–6. doi: 10.1039/d3nh00477e (PMC10962636; doi:10.1039/d3nh00477e)
Supplement: NH-009-D3NH00477E-s001 [file NH-009-D3NH00477E-s001.pdf]

Electronic Supplementary Material (ESI) for Nanoscale Horizons.
This journal is © The Royal Society of Chemistry 2024

Electronic Supplementary Information: Composition-Tunable Transition Metal Dichalcogenide Nanosheets via a Scalable, Solution-Processable Method

Rebekah A. Wells[a]*, Nicolas J. Diercks[a], Victor Boureau[b], Zhenyu Wang[c], Yanfei Zhao[c], Simon Nussbaum[a], Marc Esteve[a], Marina Caretti[a], Hannah Johnson[a,d], Andras Kis[c], Kevin Sivula[a]*

* Corresponding authors

[a] Laboratory for Molecular Engineering of Optoelectronic Nanomaterials, Institute of Chemical Sciences and Engineering, École Polytechnique Fédérale de Lausanne (EPFL), CH-1015 Lausanne, Switzerland

E-mail : Email Addresses : Rebekah.wells@epfl.ch ; Kevin.sivula@epfl.ch

[b] Interdisciplinary Center for Electron Microscopy (CIME), École Polytechnique Fédérale de Lausanne (EPFL), CH-1015 Lausanne, Switzerland

[c] Laboratory of Nanoscale Electronics and Structures, Institute of Materials Science and Engineering, École Polytechnique Fédérale de Lausanne (EPFL), CH-1015 Lausanne, Switzerland

[d] Advanced Materials Research, Toyota Motor Europe, B-1930 Zaventem, Belgium

Experimental Details

Pellet Pressing + Annealing: For alloys the desired powder amounts were weighed based on desired atomic concentration and ground together. In an effort to achieve a more homogenous mixture we manually ground the two powders together in a mortar and pestle for 2-3 minutes before pressing them into a pellet. Typically batches of 3 or 6 pellets were weighed out simultaneously, ground together, and then separated into portions for pellet pressing.

Mixed and as-received TMD powder (Sigma-Aldrich; powder < 2 µm, 99%) was pressed using a manual Beckman hydraulic pellet press, 12.5 mm diameter die, up to 10 tons and held for 5 s. It is noted that this is a standard KCl pellet press (as used for FTIR spectroscopy sample preparation) which operates by filling the die (polished plate, powder, polished plate), compressing the sandwiched powder in the die with a pushing rod, and applying the pressure via the press to the pressing rod to compact the pellet. The polished plates and pressed pellet are then gently removed from the die using the pushing rod.

For S-based materials ($MoS_{2;}$ WS_2 $Mo_{(1-x)}$; W_xS_2): Up to three 500 mg pellets were vacuum sealed in a fused quartz glass tube with 100 mg S powder ensuring the pellets were not touching to avoid fusing. The pellets were heated from room temperature to 1100 °C over 8 hours, held at 1100 °C for 48 hours, then allowed to cool naturally.

For Se-based materials ($MoSe_2$; WSe_2;$Mo_{(1-x)}W_xSe_2$): Up to three 750 mg pellets were vacuum sealed in a fused quartz glass tube with 100 mg Se powder ensuring the pellets were not touching to avoid fusing. The pellets were heated from room temperature to 1000 °C over 8 hours, held at 1000 °C for 12 hours, then allowed to cool naturally.

For S/Se-based materials ($MoS_{(2-y)}Se_y$; $WS_{(2-y)}Se_y$; $Mo_{(1-x)}W_xS_{(2-y)}Se_y$): Up to three 500 mg pellets were vacuum sealed in a fused quartz glass tube with 50 mg of S powder and 50 mg of Se powder ensuring the pellets were not touching to avoid fusing. The pellets were heated from room temperature to 1100 °C over 8 hours, held at 1100 °C for 48 hours, then allowed to cool naturally.

Electrochemical Pellet Intercalation, Exfoliation, and Size-selection: The annealed pellet was clipped with an alligator clip and placed in a 50 mL beaker with a glassy carbon counter electrode. Both electrodes were connected to a potentiostat with the TMD pellet as the working electrode (WE). A solution of tetraheptylammonium bromide (Acros Organics, 99%) in acetonitrile (Merck Millipore) was added until the pellet was submerged without liquid touching the alligator clip, and with sufficient space between electrodes to avoid contact as the WE expands. For S and S/Se-based pellets 5 mg mL^{-1} was used; for Se-based pellets 10 mg mL–1 was used.

A voltage of 10 V was applied for 24 h (WE as cathode), during which the solution begins to turn yellow at the anode, and the pellet begins to slowly expand and slough off. After the pellet has become a fluffy powder at the bottom of the beaker, the powder and remaining pellet were carefully collected and washed thoroughly with ethanol *via* vacuum filtration with a nylon filter (pore size 0.45 µm). The solid material was transferred to a 50 mL centrifuge tube with 10 mL of NMP (Acros Organics; 99+% for spectroscopy) before bath sonication (Ultrasonic bath USC T, VWR, 45 kHz) in water at 25°C for 1-2 hours.

Finally, to remove any unexfoliated bulk (including remains of the pellet, which can be reused), the solution was centrifuged for 30 min at 120 rcf using an Eppendorf centrifuge 5810 equipped with a FA-45-6-30 rotor. The top 8 mL of supernatant were collected and transferred to a new container. These processing conditions typically gave nanoflakes of mostly 1-5 layers with a lateral dimension of 500 nm to 1 µm, as has been previously established.[1] Note that additional centrifugation or filtration steps could be performed to isolate a narrower dispersion of nanosheets as needed.

Thin Film Formation: Thin films were made via a liquid/liquid interface created between deionized water and hexane (Sigma-Aldrich; >99%) using a previously-described approach, [2] slightly modified as described here (eater, hexane system, NMP for dispersion solvent).[1] Transfer to substrate was accomplished by aspirating the organic phase and then either aspirating the water phase to deposit the film onto a pre-positioned substrate (for FTO glass) or *via* a stamping method (for FET substrates) wherein the substrate is manipulated with a suction pen and pressed into the film, transferring the material onto the substrate. Films were then annealed at 200 °C for 120 min in a vacuum oven to remove excess solvent.

UV-Visible Spectroscopy: UV-Vis spectra were acquired using a Shimadzu UV 3600 spectrometer from 825-300 nm using an integrating sphere with step size of 1 nm and slit width of 5 nm. Measurements of solutions were taken using a quartz cuvette directly in transmission mode. Dispersions of nanosheets in NMP were diluted in NMP. NMP was used for a blank. Absorbance was calculated as shown in equation (1):

$$\%\text{Absorbance} = 2 - \log_{10}(\%\text{Transmission}) \tag{1}$$

Raman Spectroscopy: Raman spectra and PL spectra were obtained using a Horiba Xplora Plus Raman microscope with 532 nm radiation (40 mW). Raman spectra were acquired from 100-1800 cm^{-1} using a 100x objective, slit of 200 µm, hole of 500 µm, a grating with 2400 gr/mm, 10% filter, 10s of acquisition, and 5 accumulations. Note that the diameter of our excitation beam is large compared to average nanosheet size and the nanosheets are relatively densely packed on the substrate. This means the signal observed will be an average over many nanosheet sizes.

Photoluminescence Spectroscopy: PL spectra were obtained from 550-950 nm using a 100x objective, a grating with 600 gr/mm, slit of 200 µm, hole of 500 µm, 25% filter, 4s of acquisition, and 4 accumulations. PL spectra were normalized according to Raman signals appearing around 580 nm to adjust for material content and then to [0,1] for display clarity.

X-ray Photoelectron Spectroscopy: XPS spectra were acquired using a PHI Versa Probe II (Physical Instruments AG, Germany). Analysis was performed using a monochromatic Al-Kα X-ray source operated at 50 W. The spherical mirror analyzer was set at 45° take-off angle with respect to the sample surface. The pass energy was 46.95 eV yielding a full width at half maximum of 0.91 eV for the Ag 3d 5/2 peak.

X-Ray Diffraction: Powder XRD measurements were taken in Debye-Scherrer geometry (scanning mode) using Cu-Kα radiation on a Bruker D8 Discover Plus instrument equipped with a rotating anode and a Dectris Eiger2 500K detector. Samples were loaded into 0.5 mm borosilicate capillaries and spun during data acquisition. Lattice parameters were measured by fitting peak intensities.

FET Measurements: FET transistors were made by using LLISA to deposit 1-2 layers of TMD nanosheets onto commercially available substrates (Fraunhofer ISE): Au coated SiO_2 (210 nm) transistor substrates were used with 10 mm channel widths (W), an insulator capacitance (C_i) of 1.8 × 10–8 F., and channel lengths of 5 µm. FET measurements were carried out in a nitrogen atmosphere using a custom-built probe station and a Keithley 2612A dual-channel source measure unit. Drain voltage was scanned from 0 to 40 V with gate voltages from 0 to 40 V.

STEM EDX analyses: STEM EDX mappings and HAADF images were acquired on a Thermo Fisher Scientific Talos F200S microscope operated at 200 kV. STEM HAADF images were obtained using a probe current of 100 pA, a camera length of 77 mm and a dwell time of 1-2 µs. The elemental net count maps were processed with Velox software and the quantification used the Cliff-Lorimer method. Atomic-resolution STEM images were acquired on a Thermo Fisher Scientific Titan Themis microscope operated at 80 kV and equipped with a high-brightness field emission gun (X-FEG) and a 4-segment STEM detector used for iDPC imaging. The aberrations of the probe were corrected with a CEOS DCOR system up to the 4th order and a convergence angle of 20 mrad were used. HAADF and iDPC imaging were acquired simultaneously using a probe current of 30 pA, a camera length of 115 mm and a dwell time of 2 µs.

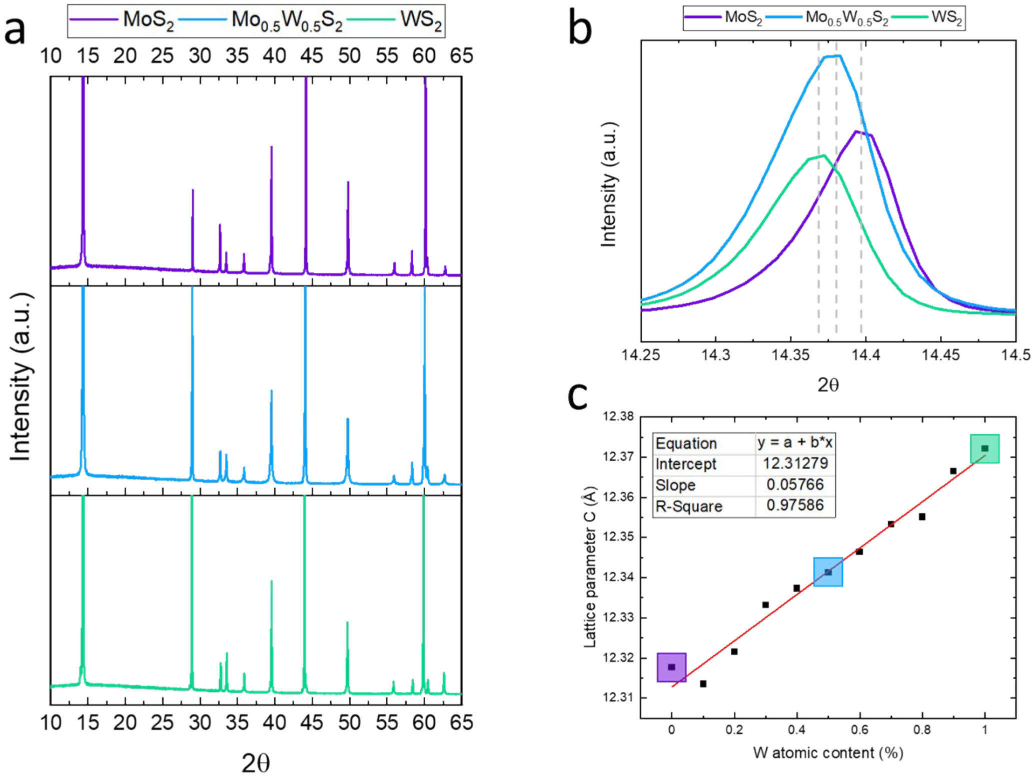

Fig. S1. Powder XRD (source: Cu-K-alpha 1) patterns of pellets made of pure MoS2 (purple), pure WS2 (green), and 1:1 MoS2:WS2 (blue). (a) Wide view shows sharp, high-intensity peaks confirming the crystallinity of the three pellets. (b) Close up of the (002) reflection shows systematic peak shifting consistent with changed lattice parameters. (c) Lattice parameter for the c-planes as a function of W atomic percent. A linear fit (red, solid line) confirms Vegard's law for alloys.

X-ray diffraction (XRD) of the pure and mixed pellets shows well defined peaks consistent with successful formation of crystalline domains. The small difference in MoS_2 and WS_2 lattice parameters (<0.5%) makes distinguishing the peaks challenging is Fig. S1a. Accordingly, a closer look at two (002) peaks at 14.35-14.40 shows a systematic shift to higher 2θ from WS_2 to $Mo_{0.5}W_{0.5}S_2$ to MoS_2 (Fig. S1b). This is consistent with an increase of lattice parameters with the incorporation of W atoms. By fitting the intensities shown in Fig. S1a, it is possible to calculate the unit cell parameters, as shown for the c-planes in Fig. S1c. Theoretical works suggest that the lattice parameters should follow Vegard's law for alloyed materials, which empirically states that combining two materials with the same crystal structure should yield a linear combination of their lattice parameters according to the composition.[3] Indeed, the change in lattice parameter shows a perfectly linear relationship as a function of W atomic percent, appearing to follow Vegard's law as theoretically predicted.

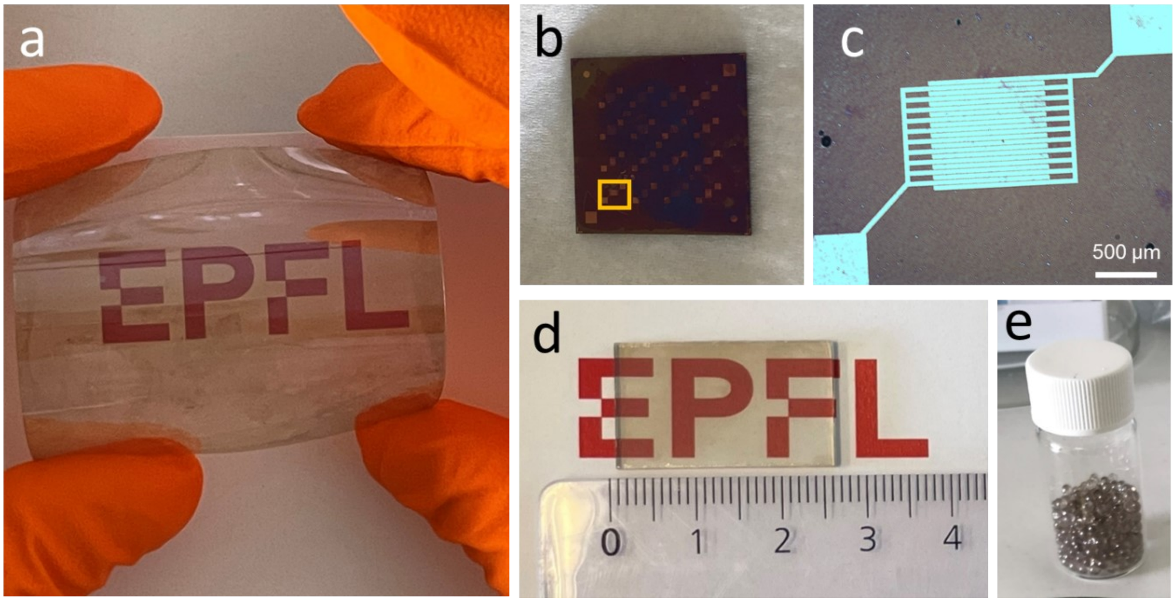

Fig. S2. Examples of alloyed TMDs coated on a variety of substrates using LLISA deposition (a-d) and drop casting (e). (a) Flexible substrate, PET, suitable for roll-to-roll applications.[4] (b) Rigid Au-bottom patterned Si/SiO_2 transistor with close up of 2.5µm gate (yellow box) shown in (c). (d) Rigid FTO-coated glass for typical photoelectrochemical testing.[1] (e) Glass beads to demonstrate atypical substrate coating.

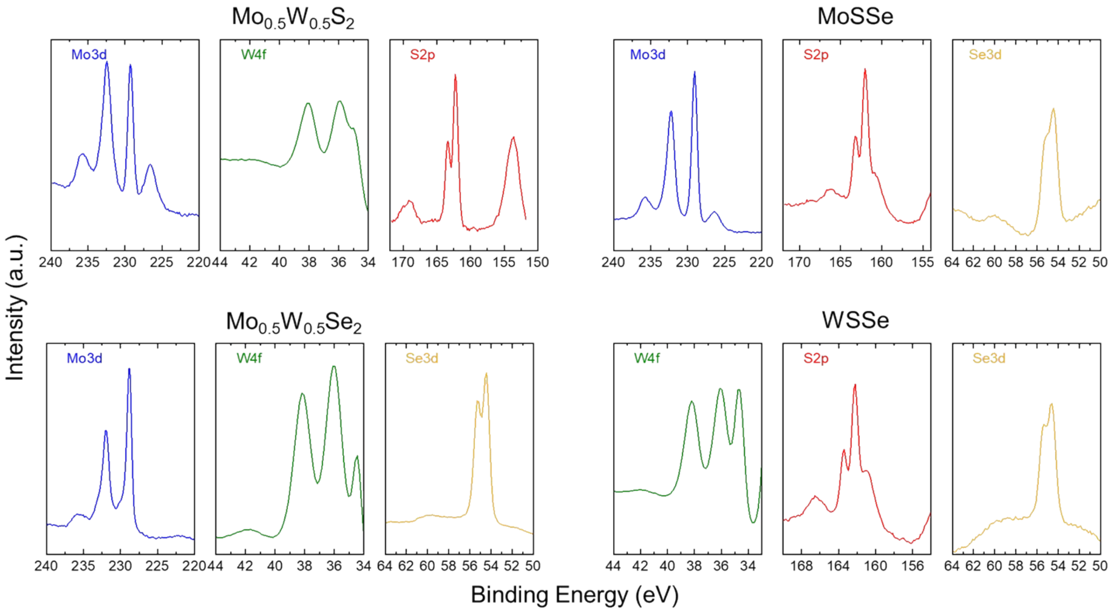

Fig. S3. Mo 3d (blue), W 4f (green), S 2p (red), and Se 3d (yellow) core level spectra for the ternary alloyed TMD nanosheets confirms the presence of the expected elements for each combination.

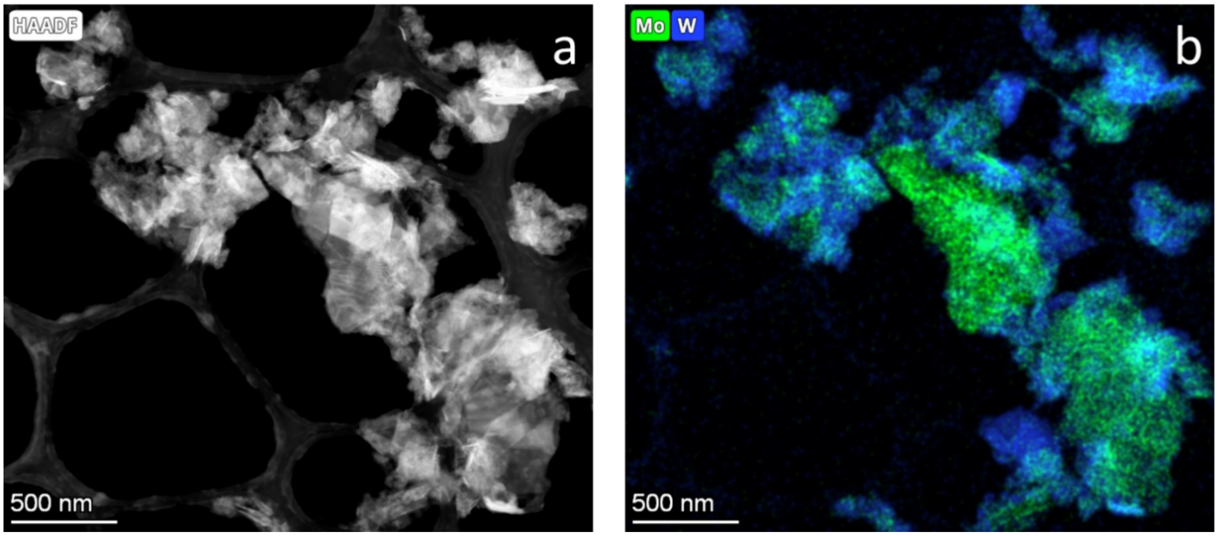

Fig. S4. STEM EDX of exfoliated nanosheets made from alloyed powders which were not mechanically ground. STEM (a) HAADF imaging and (b) EDX composite elemental mapping (net counts) for $Mo_xW_xS_2$ nanoflakes made from MoS_2 and WS_2 powders which were mixed with 1:1 atomic ratio but were not mechanically ground prior to pellet formation.

While some nanosheets show even distribution of Mo and W, indicating ternary alloy formation, others appear to be pure binary phases. This emphasizes the importance of thorough grinding of the powders before pressing them into pellets.

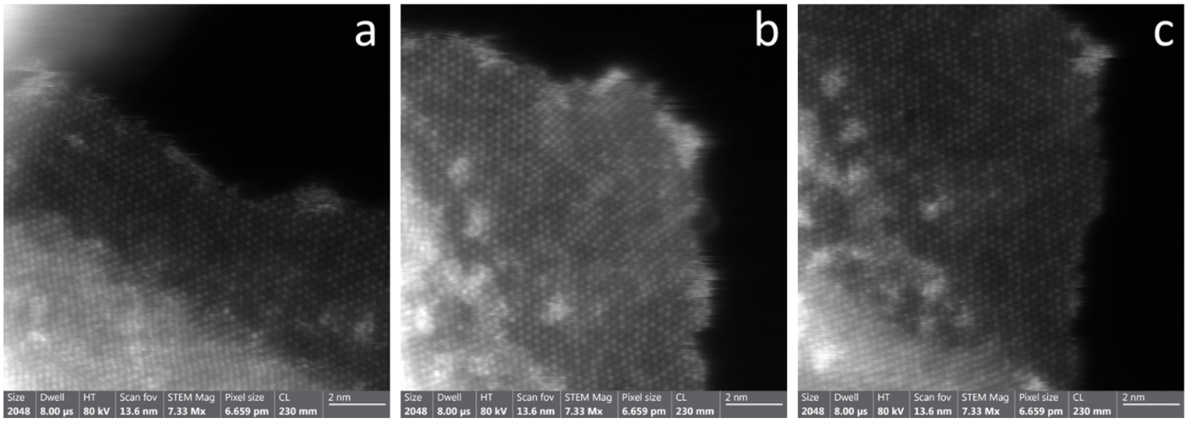

Fig. S5. Additional high-resolution STEM HAADF images for multiple $Mo_{0.5}W_{0.5}S_2$ nanosheets. Bright clusters are caused by organic contamination.

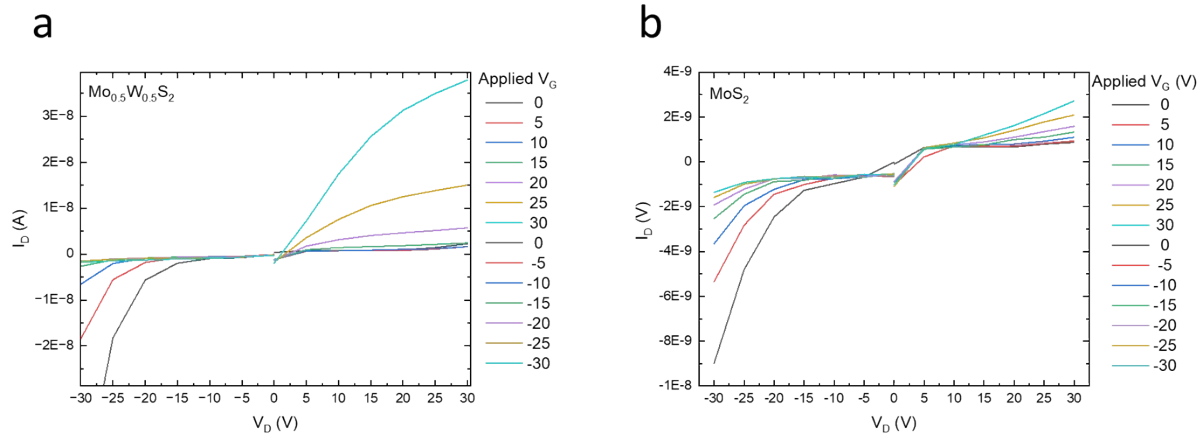

Fig. S6. Output curves for the transfer characteristics shown in Fig. 3d of the main text for FETs made from thin films of (a) $Mo_{0.5}W_{0.5}S_2$ and (b) MoS_2 nanosheets.

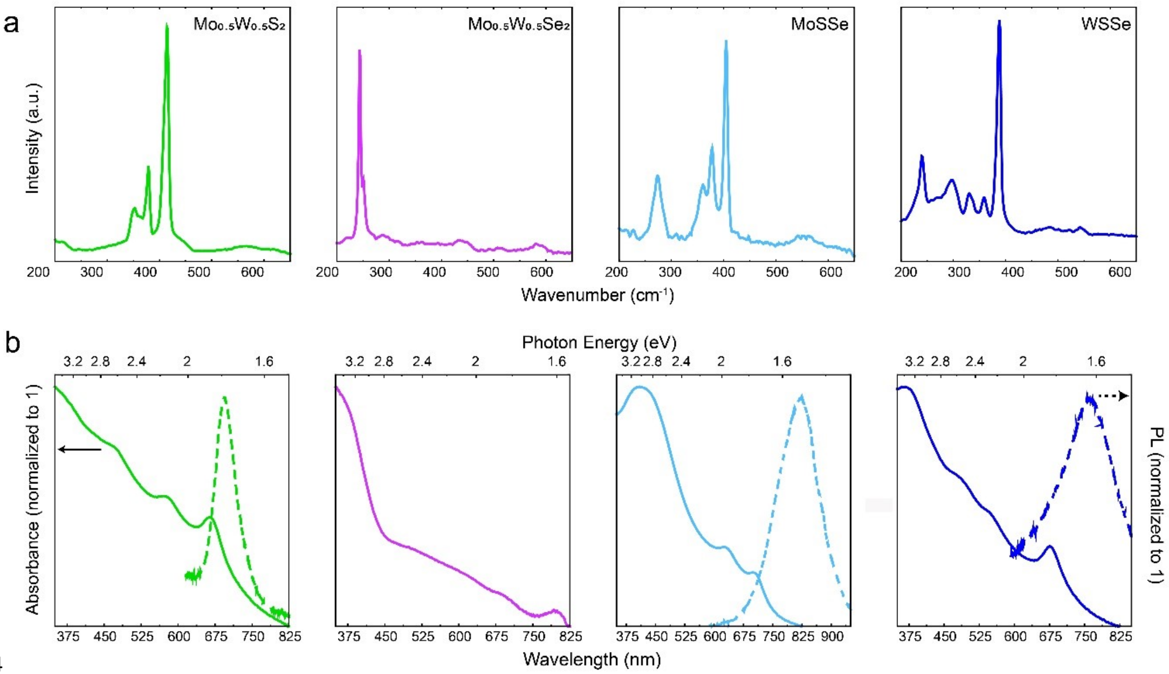

Fig. S7. Characterization of ternary alloyed nanosheets made from combinations of Mo, W, S, and Se. From left to right: $Mo_{(1-x)}W_xS_2$ (green), $Mo_{(1-x)}W_xSe_2$ (pink), MoSSe (cyan), and WSSe (blue). (a) Raman spectroscopy of thin films made of the four ternary alloys. (b) Normalized UV-Vis absorbance spectra of the nanosheet dispersions (solid lines) and normalized PL spectra of the thin films (broken lines) for the four ternary alloys.

To concretely confirm alloy formation, Raman spectra for the four ternary alloys is shown in Fig. S6a. These results show vibration modes which are distinct from the pure phases , notably the splitting of E^1_{2g} for metal alloyed nanosheets (X-Mo-X and X-W-X; X = S,Se) and the addition of modes for the chalcogen alloyed nanosheets (S-Y-S, S-Y-Se, Se-Y-Se: Y = Mo,W) consistent with monolayer CVD grown demonstrations and computational predictions.[5–13] Panel one and two (green and purple) show evidence for the typical group VI metal-alloyed TMDs, $Mo_{0.5}W_{0.5}S_2$ and $Mo_{0.5}W_{0.5}Se_2$. Notably this means that both *n*-type and *p*-type alloys can be fabricated and tuned using this method. Additionally, panel three and four (cyan and blue) confirm that chalcogen-alloyed TMDs are also attainable, finishing off the possible combinations.

The optoelectronic properties are studied using UV-Vis (solid lines) and PL (broken lines) in Fig. S6b. As with the Raman spectroscopy the UV-Vis spectra show unique absorption signals, displaying altered exciton peaks and regions of increased absorption compared to the pure TMDs. Accordingly, PL is also shifted compared to the pure materials and can be recorded for all combinations, with the exception of the $Mo_{0.5}W_{0.5}Se_2$ alloy. Given the red-shifted nature (compared to $MoSe_2$ and WSe_2) of the excitonic peak and the expected Stokes shift of the PL signal, it is possible that PL is present but not detectable in the set-up

used in this work. Another explanation could be the general instability of selenide-based materials.[14] Indeed, if too many defects are present charge recombination may occur too quickly to be detected in this very simple apparatus.

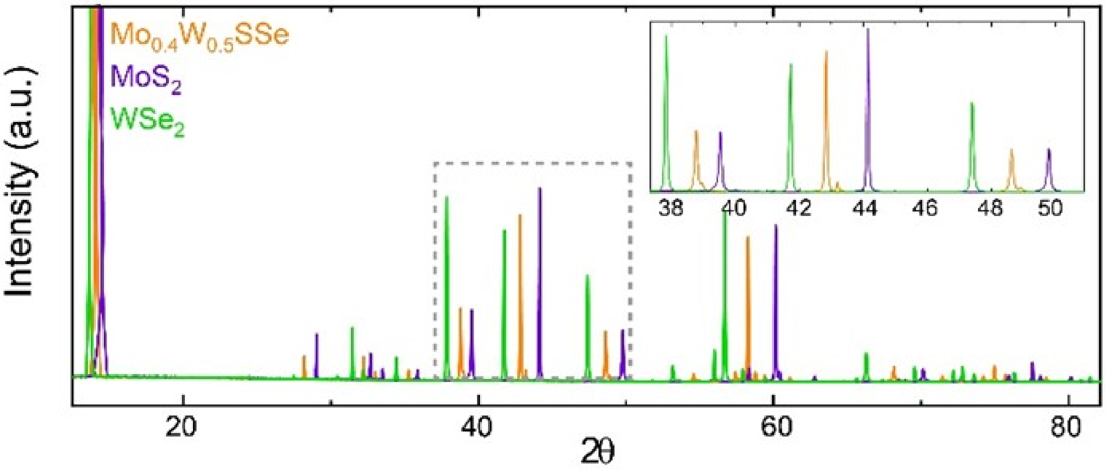

Fig. S8. Crystal lattice characterization of quaternary alloyed $Mo_{0.5}W_{0.5}SSe$ pellet. Powder XRD pattern for the alloyed pellet (orange, center peak) as well as the pure starting materials MoS_2 (purple, rightmost peak) and WS_2 (green, leftmost peak). Inset gives a closer look at reflections (1 0 3), (0 0 6), and (1 0 5) from left to right, respectively.

XRD analysis of the annealed $Mo_{0.5}W_{0.5}SSe$ pellet is shown along with XRD for the pure materials that were mixed to form the pellet. From the wide view it can be seen that all three materials give sharp peaks, consistent with the formation of crystalline domains. The inset shows a close up of the (1 0 3), (0 0 6), and (1 0 5) peaks, respectively left to right. For each reflection the quaternary alloy lies directly in the middle of the two pure materials, seemingly following Vegard's law for alloyed materials.[2] As the lattice parameters for MoS_2 and WSe_2 are quite different (>5%), a distinction is more clearly seen compared to the case of the $Mo_{0.5}W_{0.5}S_2$ pellet (described in Fig. S1).

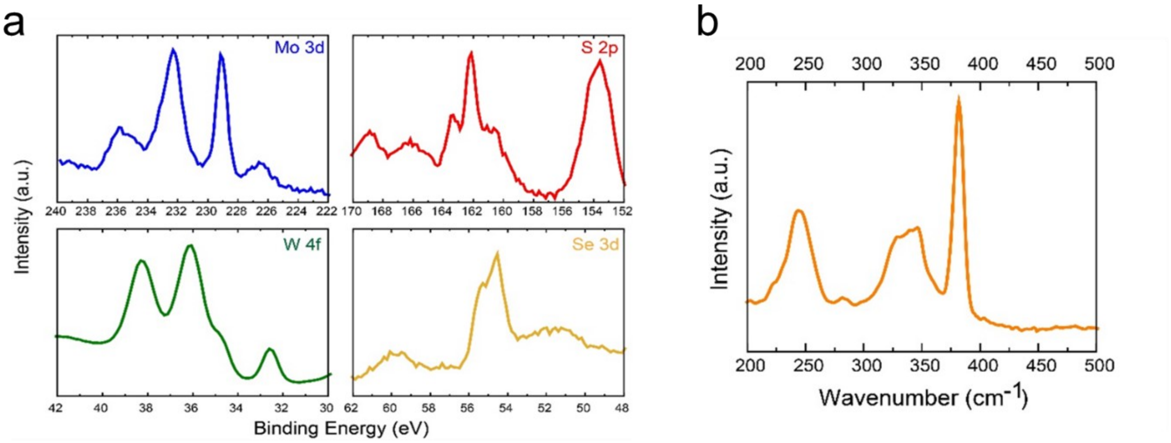

Fig. S9. Characterization for thin film of exfoliated $Mo_{0.5}W_{0.5}SSe$ nanosheets. (a) XPS spectra for a thin film of exfoliated $Mo_{0.5}W_{0.5}SSe$ for core level Mo 3d (top left, blue), W 4f (bottom left, green), S 2p (top right, red), and Se 3d (bottom right, yellow), confirming the presence of the four expected elements. (b) Raman spectrum for a thin film of exfoliated $Mo_{0.5}W_{0.5}SSe$.

The Raman spectrum displayed in Fig. S8b is complex as is expected for a material with many vibrational modes including: Mo-S-W, W-S, Mo-S, Mo-W-Se, W-S-Se, Mo-S-Se.[6] Importantly this spectrum is not only distinct from either pure material, but also from all of the ternary alloys previously discussed (see Fig. S6).

$$E_{A_{ext},Mo_{(1-x)}W_xS_2} = (1-x)E_{A_{ext},MoS_2} + xE_{A_{ext},WS_2} - bx(1-x) \tag{2}$$

Equation 2. The bowing effect parameters, where *x* is the *W* content, E_{Aext} is the energy of the excitonic peak, and *b* is the bowing parameter.[2]

Notes and References

1 R. A. Wells, M. Zhang, T.-H. Chen, V. Boureau, M. Caretti, Y. Liu, J.-H. Yum, H. Johnson, S. Kinge, A. Radenovic and K. Sivula, *ACS Nano*, 2022, **16**, 5719–5730.
2 X. Yu, M. S. Prévot, N. Guijarro and K. Sivula, *Nat. Commun.*, 2015, **6**, 7596.
3 J. Kang, S. Tongay, J. Li and J. Wu, *Journal of Applied Physics*, 2013, **113**, 143703.
4 R. A. Wells, H. Johnson, C. R. Lhermitte, S. Kinge and K. Sivula, *ACS Appl. Nano Mater.*, 2019, **2**, 7705–7712.

5 J. Park, M. S. Kim, B. Park, S. H. Oh, S. Roy, J. Kim and W. Choi, *ACS Nano*, 2018, **12**, 6301–6309.
6 S. Susarla, A. Kutana, J. A. Hachtel, V. Kochat, A. Apte, R. Vajtai, J. C. Idrobo, B. I. Yakobson, C. S. Tiwary and P. M. Ajayan, *Advanced Materials*, 2017, **29**, 1702457.
7 J.-G. Song, G. H. Ryu, S. J. Lee, S. Sim, C. W. Lee, T. Choi, H. Jung, Y. Kim, Z. Lee, J.-M. Myoung, C. Dussarrat, C. Lansalot-Matras, J. Park, H. Choi and H. Kim, *Nat. Commun.*, 2015, **6**, 7817.
8 X. Duan, C. Wang, Z. Fan, G. Hao, L. Kou, U. Halim, H. Li, X. Wu, Y. Wang, J. Jiang, A. Pan, Y. Huang, R. Yu and X. Duan, *Nano Lett.*, 2016, **16**, 264–269.
9 Q. Feng, Y. Zhu, J. Hong, M. Zhang, W. Duan, N. Mao, J. Wu, H. Xu, F. Dong, F. Lin, C. Jin, C. Wang, J. Zhang and L. Xie, *Adv. Mater.*, 2014, **26**, 2648–2653.
10 Y. Chen, D. O. Dumcenco, Y. Zhu, X. Zhang, N. Mao, Q. Feng, M. Zhang, J. Zhang, P.-H. Tan, Y.-S. Huang and L. Xie, *Nanoscale*, 2014, **6**, 2833–2839.
11 Y. Gao, J. Liu, X. Zhang and G. Lu, *J. Phys. Chem. C*, 2021, **125**, 774–781.
12 A. Hashemi, A. V. Krasheninnikov, M. Puska and H.-P. Komsa, *Phys. Rev. Mater.*, 2019, **3**, 023806.
13 A. Taghizadeh, U. Leffers, T. G. Pedersen and K. S. Thygesen, *Nat Commun*, 2020, **11**, 3011
14 C. P. Cullen, O. Hartwig, C. Ó. Coileáin, J. B. McManus, L. Peters, C. Ilhan, G. S. Duesberg and N. McEvoy, 72.
